# A Modified Technique for Two-Stage Revision in Knee PJI Treatment

**DOI:** 10.3390/jcm12237323

**Published:** 2023-11-26

**Authors:** Raffaele Iorio, Ferdinando Iannotti, Leonardo Previ, Edoardo Viglietta, Yuri Gugliotta, Federico Corsetti, Simone Fenucci, Angelo De Carli, Pier Francesco Indelli, Andrea Redler

**Affiliations:** 1 Orthopaedic Unit, S. Andrea Hospital, University of Rome, “La Sapienza” Via Di Grottarossa 1035, 00189 Rome, Italy; raffaeleiorio75@gmail.com (R.I.); leonardoprevi@gmail.com (L.P.); fedicocorsetti7@gmail.com (F.C.); simone.fenucci@gmail.com (S.F.); angelo.decarli@gmail.com (A.D.C.); 2 Division Orthopaedic Surgery, Ospedale San Paolo, 00053 Civitavecchia, Italy; ferdinandoiannotti@gmail.com; 3 Department of Orthopedic Surgery, Stanford University of School of Medicine, Stanford, CA 94063, USA; pindelli@stanford.edu

**Keywords:** periprosthetic joint infection, two-stage revision, revision total knee arthroplasty, DAPRI technique

## Abstract

Knee PJIs represent one of the most important complications after joint replacement surgery. If the prerequisites for implant retention do not subsist, the surgical treatment of these conditions is performed using one-stage and two-stage revision techniques. In this study, an implemented two-stage revision technique was performed, adopting antibiotic calcium sulfate beads and tumor-like debridement guided by methylene blue, such as described for the DAPRI technique. The aim of the present study is to compare the implemented two-stage revision technique with the standard technique in order to assess its effectiveness. Methods. Twenty patients affected by knee PJIs were prospectively enrolled in the study and underwent an implemented two-stage revision technique (Group A). Data collected and clinical results were compared with a matched control group treated with a standard two-stage technique (Group B). For each patient, the time of the reimplantation and length of antibiotic systemic therapy were recorded. Each patient underwent routine laboratory tests, including inflammatory markers. Results. In Group A and in Group B, inflammatory markers normalized at 6.5 ± 1.1. weeks and 11.1 ± 2.3 weeks, respectively (*p* < 0.05). Also, the difference in length of antibiotic therapy and time to reimplantation were significantly shorter in Group A (*p* < 0.05). No recurrence of infection was found in Group A at the last follow-up. Discussion. The implemented two-stage revision technique demonstrated a faster normalization of inflammatory markers, as well as a decrease in reimplantation time and duration of antibiotic therapy, compared to the traditional technique. The use of calcium sulfate antibiotic beads and tumor-like debridement seems to improve the results and reduce the time of healing. Conclusion. The implemented two-stage revision technique seems to improve the results and reduce the time of healing. This leads to a more rapid and less stressful course for the patient, as well as a reduction in health care costs.

## 1. Introduction

### 1.1. Periprosthetic Joint Infection

Hip and knee prosthetic surgeries have become some of the most successful procedures in orthopedic surgery in recent decades [1]. They have excellent functional outcomes and provide a significant improvement in overall quality of life for patients, but as the number of primary interventions has increased, the number of failures and revisions has grown [2,3,4]. Moreover, the longer the implanted prosthesis is expected to last, the greater the cumulative risk of developing complications and infection [5].

Periprosthetic joint infection (PJI) represents one of the most important and feared complications after joint replacement surgery [6]; the incidence varies between 0.5 and 2.2% [7,8]. PJI is the most common cause of total knee arthroplasty (TKA) failure and revision surgery [9,10].

PJI has a dramatic impact on the patient’s life; in fact, septic revision surgery, regardless of the technique, correlates with 90-day and 1-year mortality rates 5 times higher than aseptic surgery and reaches a mortality rate after 5 years that is comparable to those of oncological patients [11].

The management of PJI is still controversial and requires a complex therapeutic approach, prolonged antibiotic therapy, and the use of various surgical techniques [5]. Two-stage revision arthroplasty is still considered the gold standard for the surgical treatment of PJI [12]; however, the impact of revision surgery is impressive. The period between the two stages, which can last from 6 weeks to several months, in particular, has a significant negative impact on mobility and usually confers a subsequent dependency on the patient. Other significant negative consequences reported include the burden of systemic antibiotic therapy, changing interfamily dynamics, and a strong psychological impact [13,14].

Among the greatest problems in PJI is the presence of biofilm, which enables the responsible pathogens to remain on the implant surface, making them resistant to most systemic antibiotic therapy [15,16,17].

### 1.2. The Implemented Technique

The classical PJI treatment alternatives include “debridement, antibiotics, and implant retention” (DAIR); single-stage revision; and double-stage revision [18].

The “debridement, antibiotic pearls and retention of the Implant” (DAPRI) protocol represents a surgical technique alternative to DAIR [19,20]. It can save the implant and consists of aggressive soft-tissue debridement guided by methylene blue, including culture sampling, extensive pulse lavage, insert replacement, and the use of local calcium sulfate antibiotic beads, plus systemic antibiotic therapy. When it is not possible to retain the implant, one- or two-stage exchange surgery is used. 

In this study, an implemented two-stage revision technique was performed in knee PJI, adopting antibiotic calcium sulfate beads and tumor-like debridement guided by methylene blue, such as previously described for the DAPRI technique [21].

The aim of the present study is to assess the time and rate of infection healing with the implemented two-stage revision technique compared with the standard two-stage technique. The hypothesis is that the implemented two-stage technique can allow for a faster infection resolution.

## 2. Materials and Methods

### 2.1. Study Design and Population

Twenty patients suffering from hip or knee PJIs were prospectively enrolled in the study from June 2019 to December 2021 and received an implanted two-stage revision technique.

The study protocol was approved by the local ethics committee. Written informed consent to participate was obtained from each patient.

Inclusion criteria were patients affected by knee PJI diagnosed according to 2018 Consensus Meeting on Musculoskeletal Infection criteria [22]; aged 18 years or above; diagnosis made more than 4 weeks from first implantation or symptoms onset for more than a week; requiring revision surgery; positive preoperative culture and microbic identification; sensitivity of the microorganism to antibiotic therapy. Exclusion criteria were polymicrobial infection, allergy to any study material, immunosuppressed patient, and generalized sepsis. 

All patients included in the study underwent an implemented two-stage revision technique. Data collected and clinical results were compared with a match control group, whose data were collected for another study in the past 3 years from the beginning of the present study, in which patients were treated according to the standard technique.

### 2.2. Standard Two-Stage Revision Surgical Technique

The two-stage revision procedure was used for Group B patients. In the first stage, the previous surgical scars were used to approach the joint. All implanted materials were removed, and radical debridement, including soft tissues, membranes, and potentially infected or devitalized tissue, was provided. A minimum of 3 to 5 or 6 periprosthetic intraoperative tissue samples or the explanted prosthesis were sent for aerobic and anaerobic cultures to obtain a definitive diagnosis. An extensive lavage with high-pressure pulsatile lavage system using at least 6 L of normal saline was performed. A Cement spacer was positioned following the implant removal. The antibiotic added to the spacer’s cement was chosen on preoperative culture and sensitivity testing. During interim period between the two stages, patients underwent a regimen of 4–6 weeks of intravenous antibiotics, followed by discontinuation of the antibiotics for a period of 2–8 weeks prior to the second stage. Antibiotic therapy was tailored to microorganism antimicrobial sensitivity. Empiric therapy should be started if the organism or sensitivities are unknown. The decision to proceed with prosthesis implantation was determined by clinical evaluation and resolution of blood markers.

### 2.3. Implemented Two-Stage Revision Surgical Technique

Patients included in Group A underwent the implemented two-stage revision surgical technique. During the first stage, methylene blue-guided debridement was performed similarly to the DAPRI technique [21]. Prior to skin incision, an arthrocentesis was performed, and the aspirated material was sent for cultural analysis; with the same needle, 50 mL of diluted (0.1%) methylene blue (40 mL normal saline and 10 mL of 0.5% methylene blue solution) was then injected into the knee joint. Methylene blue is known to stain bacterial biofilm and all tissues in the effective joint space [23,24,25]. After injection, the knee underwent multiple rounds of flexion and extension to facilitate the intra-articular distribution of the staining dye. An arthrocentesis was then performed to remove the excessive dye. After the arthrotomy was performed, all stained soft tissues were removed, as these tissues had been in contact with the infected intra-articular space. The implant was then removed. Five soft tissue culture samples from different stained areas were sent for standard microbiological studies (aerobic, anaerobic, and fungal exams). A pulsed jet lavage with 5 L of 0.9% saline and 150 mL of sterile 10% povidone-iodine was used during surgery. 

A patient-specific, antibiotic-loaded cement articulated spacer was implanted (Cemex, Tecres S.p.A., Sommacampagna, Italy), according to the pre-operative antibiogram.

Irrigation with 0.65% diluted povidone iodine solution is then performed by mixing 35 mL of sterile 10% povidone-iodine with 500 mL of 0.9% saline. Further irrigation of the joint was performed using saline pulse irrigation. Thereafter, calcium sulfate antibiotic-filled beads (Stimulan; Biocomposites Ltd., Keele, UK) were prepared by mixing a 10 mL kit of PG-CSH (Stimulan; Biocomposites Ltd., Keele, UK) with antibiotics selected according to the antibiogram obtained at the time of micro-organism identification. Alternatively, 1000 mg of vancomycin hydrochloride powder and 320 mg of gentamycin were used. A smooth paste was first formed mixing all components for 60 s and pressed into 4.8 mm diameter hemispherical cavities in a flexible mold. The beads usually become hard and ready for implantation after resting between 30 and 60 min. The beads are normally reabsorbed over 4–6 weeks and allow for a continuous local elution of the appropriate antibiotic.

### 2.4. Postoperative Protocol 

Postoperative systemic antibiotic therapy was prescribed by the infectivologist from preoperative and intraoperative culture results.

All patients followed the same postoperative rehabilitation protocol. 

Time to reimplantation was decided by a multidisciplinary team according to inflammatory markers normalization and clinical condition of the patients.

Each patient underwent weekly routine laboratory tests, including inflammatory markers (total white blood cell (WBC), C-reactive protein (CRP), Erythrocyte Sedimentation Rate (ESR), D-Dimer, and procalcitonin) and clinical control after surgery. Clinical examination focused on looking for swelling, secretion, or other infection signs. 

Reimplantation surgery was performed at least after 4 week of systemic antibiotic therapy and after a wash-out period from antibiotic therapy of 2 weeks (it is the same protocol adopted in the standard technique). 

For each patient, time of the reimplantation and length of antibiotic systemic therapy were recorded. Patients were evaluated every month for the first 6 months and at last follow-up at 1 year after the second stage surgery. Each patient received clinical and radiological evaluation at 1, 3, and 6 months after the second stage procedure, including clinical examination, inflammatory markers testing, and radiographs.

### 2.5. Statistical Analysis 

A matched-pair analysis was applied to minimize the impact of extraneous factors and any potential selection bias. A propensity score was calculated for each patient using the following parameters: age at the time of two-stage revision surgery (<80 years or >80 years), PJI site, body mass index, sex (male or female), time from diagnosis to surgery (<3 or >3 days), and patient immunity status according to McPherson [26]. 

Then, each patient who received the implemented two-stage surgery was matched with a patient who underwent the standard two-stage revision surgery, according to the nearest corresponding propensity score [27].

A descriptive analysis was performed on all variables of interest. The statistical analysis was performed using the Student’s *t*-test; the level of statistical significance was set at 0.05, and the confidence interval was set at 95%.

## 3. Results

A total of 20 patients (12 male and 8 female) were enrolled in the study. 

There was no statistically significant difference between the study group (Group A) and match paired group (Group B) regarding demographic parameters, host immunity status according to McPherson, and the type of microorganisms responsible (Table 1).

The most represented bacterium was Staphylococcus Aureus, followed by Coagulase-Negative Staphylococci (Table 2).

Normalized inflammatory biochemical markers were reached in all patients in Group A at a mean of 6.5 ± 1.1 weeks and 11.1 ± 2.3 weeks in Group B (*p* < 0.05); 

The length of antibiotic therapy was 7.1 ± 1.8 and 12 ± 1.3 weeks in Groups A and B, respectively (*p* < 0.05).

The time to reimplantation, after a 2-week wash out of antibiotic therapy, was lower in the study group (12.5 ± 2.4 vs. 18.4 ± 3.3 weeks, *p* < 0.05) (Table 3).

Intraoperative culture samples at the time of reimplantation were negative for all the patients in both groups. A case of reinfection was detected in Group B, treated with a second standard two-stage revision surgery because of PJI recurrence after 9 months from the revision. 

No other reoperations and no signs of infection were detected in both groups at the latest follow-up.

## 4. Discussion

The main finding of the present study is that the implemented two-stage revision technique seems to accelerate the final reimplantation, compared to the standard technique. In this study, the implemented two-stage revision technique proved to achieve quicker healing from PJI compared to the standard technique. The time to normalization of inflammatory markers and time to reimplantation were significantly shorter. Thus, the hypothesis was confirmed. The implemented technique was successful in all cases studied, although the authors are aware of the paucity of sample size. 

Most human bacterial infections involve biofilm-producing microorganisms, and the current literature agrees that biofilm is the main actor in PJI’s development [28,29]. Biofilm protects bacteria from immune response and antibiotic therapy and promotes the exchange of genetic material [17]. Biofilm removal is mandatory for success with PJI’s treatment [28,30], so total removal of the infected tissue is key during surgery, but it is difficult to differentiate between good and infected tissue; completeness of removal mostly depends on surgeon experience, and it is not reproducible. Methylene blue-guided debridement helps this practice as it stains biofilm, infected cells, and non-vital tissue without affecting the culture exam [23]. 

The calcium sulfate beads represent a considerable innovation as they create an environment hostile to bacteria and biofilm synthesis [31,32,33]; Knecht [34] and Cooper [35] demonstrated that antibiotic-loaded calcium sulfate beads can guarantee a high intra-articular antibiotics concentration, specific and prolonged (up to 40 days) without reaching toxic levels, which systemic therapy cannot achieve. Furthermore, they are normally absorbed within 6 weeks, leaving no foreign body surface for new bacterial colonization [36]. Abosala and Ali [37], in their systematic review, showed the use of antibiotic-loaded calcium sulfate beads delivering high doses of antibiotics locally and their role as an adjuvant in revision surgery.

The main disadvantages of the two-stage revision technique compared to the single-stage technique are certainly the length of the overall treatment and the patient’s quality of life between the two surgical stages [13,38]. 

However, the use of the single-stage revision surgery technique is still controversial because of the associated high recurrence rate of postoperative infection, and even though an increasing number of surgeons choose to use this technique, its effectiveness needs to be further explored [39,40]. 

The right time for reimplantation in the two-stage revision technique is the subject of great debate [41]. The current literature has reported that the time to reimplantation ranges from a few weeks to several months [42]. Some authors have reported an increased re-infection risk associated with prolonged time intervals between the two stages. Fu [43] reported that a spacer maintained for more than 16 weeks correlated with poor outcomes and a higher failure rate; in this consideration, the implemented two-stage revision technique can certainly be helpful in reaching the best outcome for the patient. Aalirezaie [44] found that patients who underwent the second stage at >26 weeks had twice the risk of failure than patients reimplanted within <26 weeks. In his study, Vielgut established that the optimal retention time of the spacer is less than 12 weeks [45], while Borsinger observed that retention longer than 18 weeks was associated with a higher failure rate [46]. Moreover, in addition to the reinfection risk, prolonged spacer retention length is associated with worse clinical and functional outcomes [47,48].

The shortening of the two-stage treatment achieved with the implemented technique implies an important advantage for life patients and has socioeconomic benefits that may mitigate the cost of the beads. In fact, the number of medical examinations, tests, and overall antibiotics prescribed is also reduced. The shortening of antibiotic systemic therapy can also reduce the total toxicity and side effects of the treatment.

Furthermore, in this preliminary study, the implemented technique was adopted for each patient included, but it is possible that a more targeted use could increase the benefits of the technique.

The authors believe that some patients could be more suitable for this treatment (such as in case of antibiotic resistance, major comorbidities, and multi-germ infections), but further studies are needed to investigate this further. It would also be interesting to analyze the implemented technique in the context of single-stage revision surgery.

Even if, in the authors’ experience, no complications have occurred in patients who underwent the implemented two-stage revision surgery, calcium sulfate beads are not free from complications; the main side effects reported in the literature are the presence of a copious exudate from the wound (that may require surgical intervention), heterotopic ossifications [49], and transient hypercalcemia [50].

The present study has some limitations. First of all, it is not a randomized control trial, although the patients were recruited prospectively. Furthermore, the protocol for antibiotic administration was not standardized but tailored to the patient; the different microorganisms isolated required different antibiotics. The study was a single-center study performed in an academic tertiary referral center, and the number of patients was not large. Another limitation was the lack of PROMs correlation between groups, as the study was focused on length of therapy and trends in markers of inflammation. Further studies should be performed to investigate the clinical outcomes of the implemented two-stage technique. The results obtained are scientifically valid and clinically relevant and may be of help to other surgeons facing these demanding and potentially catastrophic events.

## 5. Conclusions

The major conclusion of the present study is that the implemented two-stage revision technique is probably the best treatment solution in patients affected by knee PJI and in which pre-operative cultures are available. The authors did not record any major complications but are aware that the sample studied is limited. The shortening of the inter-stage period seems to be the biggest benefit for the patients and their quality of life. This leads to a more rapid and less stressful course for the patient, as well as a reduction in health care costs.

Further studies are needed to investigate the potential of the implemented two-stage technique, and its role should also be studied in the context of the one-stage treatment.

## Figures and Tables

**Table 1:** Demographic data.

	Group A, *n* = 20	Group B, *n* = 20	*p*
Age	76 ± 10.75	75.6 ± 10.2	
Male	12 (60%)	10 (50%)	
Famale	8 (40%)	10 (50%)	
BMI	27 ± 5.2	28 ± 4.8	
McPherson			>0.05
A	4 (20%)	6 (30%)	
B	8 (40%)	6 (30%)	
C	8 (40%)	8 (40%)	

**Table 2:** Microbiological data.

	Group A, *n* = 20	Group B, *n* = 20
Bacteria		
MSSA	7 (35%)	6 (30%)
MRSA	6 (30%)	5 (25%)
CN Staf.	4 (20%)	6 (30%)
Streptococcus	1 (5%)	2 (10%)
Enterococcus	2 (10%)	0 (0%)
*E. coli*	0 (0%)	1 (5%)

MSSA: Methicillin-sensitive Staphylococcus Aureus; MRSA: Methicillin-resistant Staphylococcus Aureus; CN Staf.: Coagulase-Negative Staphylococci. *E. coli*: *Escherichia coli*.

**Table 3:** Results.

	Group A	Group B	*p*
Time to normalization of inflammatory markers	6.5 ± 1.1 week	11.1 ± 2.3 week	0.0001
Length of antibiotic systemic therapy	7.1 ± 1.8 week	12 ± 1.3 week	0.0380
Time to reimplantation	12.5 ± 2.4 week	18.4 ± 3.3 week	0.0011

## Data Availability

The data presented in this study are available on reasonable requests from the corresponding author. The data are not publicly available due to privacy.

## References

[1] Ethgen O., Bruyère O., Richy F., Dardennes C., Reginster J.-Y. (2004). Health-Related Quality of Life in Total Hip and Total Knee Arthroplasty. A Qualitative and Systematic Review of the Literature. J. Bone Jt. Surg. Am..

[2] Sharkey P.F., Lichstein P.M., Shen C., Tokarski A.T., Parvizi J. (2014). Why Are Total Knee Arthroplasties Failing Today—Has Anything Changed After 10 Years?. J. Arthroplast..

[3] Klem N.-R., Kent P., Smith A., Dowsey M., Fary R., Schütze R., O’Sullivan P., Choong P., Bunzli S. (2020). Satisfaction after Total Knee Replacement for Osteoarthritis Is Usually High, but What Are We Measuring? A Systematic Review. Osteoarthr. Cartil. Open.

[4] Finch D.J., Martin B.I., Franklin P.D., Magder L.S., Pellegrini V.D. (2020). Patient-Reported Outcomes Following Total Hip Arthroplasty: A Multicenter Comparison Based on Surgical Approaches. J. Arthroplast..

[5] Alrayes M.M., Sukeik M. (2023). Two-Stage Revision in Periprosthetic Knee Joint Infections. World J. Orthop..

[6] Shahi A., Tan T.L., Chen A.F., Maltenfort M.G., Parvizi J. (2017). In-Hospital Mortality in Patients With Periprosthetic Joint Infection. J. Arthroplast..

[7] Davidson D.J., Spratt D., Liddle A.D. (2019). Implant Materials and Prosthetic Joint Infection: The Battle with the Biofilm. EFORT Open Rev..

[8] Zimmerli W., Trampuz A., Ochsner P.E. (2004). Prosthetic-Joint Infections. N. Engl. J. Med..

[9] Beam E., Osmon D. (2018). Prosthetic Joint Infection Update. Infect. Dis. Clin. N. Am..

[10] Koh C.K., Zeng I., Ravi S., Zhu M., Vince K.G., Young S.W. (2017). Periprosthetic Joint Infection Is the Main Cause of Failure for Modern Knee Arthroplasty: An Analysis of 11,134 Knees. Clin. Orthop. Relat. Res..

[11] Zmistowski B., Karam J.A., Durinka J.B., Casper D.S., Parvizi J. (2013). Periprosthetic Joint Infection Increases the Risk of One-Year Mortality. J. Bone Jt. Surg..

[12] Kini S.G., Gabr A., Das R., Sukeik M., Haddad F.S. (2016). Two-Stage Revision for Periprosthetic Hip and Knee Joint Infections. Open Orthop. J..

[13] Moore A.J., Blom A.W., Whitehouse M.R., Gooberman-Hill R. (2015). Deep Prosthetic Joint Infection: A Qualitative Study of the Impact on Patients and Their Experiences of Revision Surgery. BMJ Open.

[14] Anis H.K., Warren J.A., Klika A.K., Navale S.M., Zhou G., Barsoum W.K., Higuera C.A., Piuzzi N.S. (2022). Greater Prevalence of Mental Health Conditions in Septic Revision Total Knee Arthroplasty: A Call to Action. J. Knee Surg..

[15] Del Pozo J.L. (2018). Biofilm-Related Disease. Expert. Rev. Anti Infect. Ther..

[16] Romanò C.L., Romanò D., Morelli I., Drago L. (2016). The Concept of Biofilm-Related Implant Malfunction and “Low-Grade Infection”. A Modern Approach to Biofilm-Related Orthopaedic Implant Infections.

[17] Arciola C.R., Campoccia D., Montanaro L. (2018). Implant Infections: Adhesion, Biofilm Formation and Immune Evasion. Nat. Rev. Microbiol..

[18] Risitano S., Sabatini L., Atzori F., Massè A., Indelli P.F. (2018). Static Antibiotic Spacers Augmented by Calcium Sulphate Impregnated Beads in Revision TKA: Surgical Technique and Review of Literature. J. Orthop..

[19] Ghirardelli S., Fidanza A., Prati P., Iannotti F., Indelli P.F. (2020). Debridement, Antibiotic Pearls, and Retention of the Implant in the Treatment of Infected Total Hip Arthroplasty. HIP Int..

[20] Indelli P.F., Ghirardelli S., Valpiana P., Bini L., Festini M., Iannotti F. (2023). Debridement, Antibiotic Pearls, and Retention of the Implant (DAPRI) in the Treatment of Early Periprosthetic Joint Infections: A Consecutive Series. Pathogens.

[21] Calanna F., Chen F., Risitano S., Vorhies J.S., Franceschini M., Giori N.J., Indelli P.F. (2019). Debridement, Antibiotic Pearls, and Retention of the Implant (DAPRI): A Modified Technique for Implant Retention in Total Knee Arthroplasty PJI Treatment. J. Orthop. Surg..

[22] Schwarz E.M., Parvizi J., Gehrke T., Aiyer A., Battenberg A., Brown S.A., Callaghan J.J., Citak M., Egol K., Garrigues G.E. (2019). 2018 International Consensus Meeting on Musculoskeletal Infection: Research Priorities from the General Assembly Questions. J. Orthop. Res..

[23] Shaw J.D., Miller S., Plourde A., Shaw D.L., Wustrack R., Hansen E.N. (2017). Methylene Blue–Guided Debridement as an Intraoperative Adjunct for the Surgical Treatment of Periprosthetic Joint Infection. J. Arthroplast..

[24] Coulthwaite L., Verran J. (2009). Evaluation of in Vivo Denture Plaque Assessment Methods. Br. Dent. J..

[25] Parry J.A., Karau M.J., Kakar S., Hanssen A.D., Patel R., Abdel M.P. (2017). Disclosing Agents for the Intraoperative Identification of Biofilms on Orthopedic Implants. J. Arthroplast..

[26] Coughlan A., Taylor F. (2020). Classifications in Brief: The McPherson Classification of Periprosthetic Infection. Clin. Orthop. Relat. Res..

[27] Mitchell A.F.S., Krzanowski W.J. (1985). The Mahalanobis Distance and Elliptic Distributions. Biometrika.

[28] Römling U., Balsalobre C. (2012). Biofilm Infections, Their Resilience to Therapy and Innovative Treatment Strategies. J. Intern. Med..

[29] Connaughton A., Childs A., Dylewski S., Sabesan V.J. (2014). Biofilm Disrupting Technology for Orthopedic Implants: What’s on the Horizon?. Front. Med..

[30] Gbejuade H.O., Lovering A.M., Webb J.C. (2015). The Role of Microbial Biofilms in Prosthetic Joint Infections. Acta Orthop..

[31] Roberts R., McConoughey S.J., Calhoun J.H. (2014). Size and Composition of Synthetic Calcium Sulfate Beads Influence Dissolution and Elution Rates in Vitro. J. Biomed. Mater. Res. B Appl. Biomater..

[32] Howlin R.P., Winnard C., Frapwell C.J., Webb J.S., Cooper J.J., Aiken S.S., Stoodley P. (2016). Biofilm Prevention of Gram-Negative Bacterial Pathogens Involved in Periprosthetic Infection by Antibiotic-Loaded Calcium Sulfate Beads in Vitro. Biomed. Mater..

[33] Aiken S.S., Cooper J.J., Florance H., Robinson M.T., Michell S. (2015). Local Release of Antibiotics for Surgical Site Infection Management Using High-Purity Calcium Sulfate: An In Vitro Elution Study. Surg. Infect..

[34] Knecht C.S., Moley J.P., McGrath M.S., Granger J.F., Stoodley P., Dusane D.H. (2018). Antibiotic Loaded Calcium Sulfate Bead and Pulse Lavage Eradicates Biofilms on Metal Implant Materials in Vitro. J. Orthop. Res..

[35] Cooper J., Florance H., McKinnon J., Laycock P., Aiken S. (2016). Elution Profiles of Tobramycin and Vancomycin from High-Purity Calcium Sulphate Beads Incubated in a Range of Simulated Body Fluids. J. Biomater. Appl..

[36] Azzam K.A., Seeley M., Ghanem E., Austin M.S., Purtill J.J., Parvizi J. (2010). Irrigation and Debridement in the Management of Prosthetic Joint Infection: Traditional Indications Revisited. J. Arthroplast..

[37] Abosala A., Ali M. (2020). The Use of Calcium Sulphate Beads in Periprosthetic Joint Infection, a Systematic Review. J. Bone Jt. Infect..

[38] Blom A.W., Lenguerrand E., Strange S., Noble S.M., Beswick A.D., Burston A., Garfield K., Gooberman-Hill R., Harris S.R.S., Kunutsor S.K. (2022). Clinical and Cost Effectiveness of Single Stage Compared with Two Stage for Hip Prosthetic Joint Infection (INFORM): Pragmatic, Parallel Group, Open, Randomised Controlled Trial. BMJ.

[39] Belay E.S., Danilkowicz R., Bullock G., Wall K., Garrigues G.E. (2020). Single-Stage versus Two-Stage Revision for Shoulder Periprosthetic Joint Infection: A Systematic Review and Meta-Analysis. J. Shoulder Elbow Surg..

[40] Masters J.P.M., Smith N.A., Foguet P., Reed M., Parsons H., Sprowson A.P. (2013). A Systematic Review of the Evidence for Single Stage and Two Stage Revision of Infected Knee Replacement. BMC Musculoskelet. Disord..

[41] Sousa R., Carvalho A., Soares D., Abreu M.A. (2023). Interval between Two-Stage Exchanges: What Is Optimal and How Do You Know?. Arthroplasty.

[42] Aalirezaie A., Abolghasemian M., Busato T., Dennis D., Ghazavi M., Holst D.C., Kelly M., Kissin Y.D., Kuijpers M., Lange J. (2019). Hip and Knee Section, Treatment, Two-Stage Exchange: Proceedings of International Consensus on Orthopedic Infections. J. Arthroplast..

[43] Fu J., Ni M., Li H., Li X., Chai W., Zhou Y., Hao L., Chen J. (2018). The Proper Timing of Second-Stage Revision in Treating Periprosthetic Knee Infection: Reliable Indicators and Risk Factors. J. Orthop. Surg. Res..

[44] Aali Rezaie A., Goswami K., Shohat N., Tokarski A.T., White A.E., Parvizi J. (2018). Time to Reimplantation: Waiting Longer Confers No Added Benefit. J. Arthroplast..

[45] Vielgut I., Schwantzer G., Leithner A., Sadoghi P., Berzins U., Glehr M. (2021). Successful Two-Stage Exchange Arthroplasty for Periprosthetic Infection Following Total Knee Arthroplasty: The Impact of Timing on Eradication of Infection. Int. J. Med. Sci..

[46] Borsinger T.M., Resnick C.T., Werth P.M., Schilling P.L., Moschetti W.E. (2022). Does Time to Reimplantation After Explant for Prosthetic Joint Infection Influence the Likelihood of Successful Outcomes at 2 Years?. J. Arthroplast..

[47] Golgelioglu F., Oguzkaya S., Misir A., Guney A. (2020). The Effect of Time Spent with a Dynamic Spacer on Clinical and Functional Outcomes in Two-Stage Revision Knee Arthroplasty. Indian. J. Orthop..

[48] Kim D.Y., Seo Y.C., Kim C.W., Lee C.R., Jung S.H. (2022). Factors Affecting Range of Motion Following Two-Stage Revision Arthroplasty for Chronic Periprosthetic Knee Infection. Knee Surg. Relat. Res..

[49] McPherson M.F.E., Dipane B.M., Sherif M.S. (2013). Dissolvable Antibiotic Beads in Treatment of Periprosthetic Joint Infection and Revision Arthroplasty—The Use of Synthetic Pure Calcium Sulfate (Stimulan®) Impregnated with Vancomycin & Tobramycin. Reconstr. Rev..

[50] Kallala R., Haddad F.S. (2015). Hypercalcaemia Following the Use of Antibiotic-Eluting Absorbable Calcium Sulphate Beads in Revision Arthroplasty for Infection. Bone Jt. J..

